# Supplementary material for: Genome replication in asynchronously growing microbial populations
Source: PLoS Comput Biol. 2024 Jan 5;20(1):e1011753. doi: 10.1371/journal.pcbi.1011753 (PMC10796026; doi:10.1371/journal.pcbi.1011753)
Supplement: S1 Appendix — (PDF) [file pcbi.1011753.s001.pdf]

Supporing Information S1 Appendices: Genome replication in asynchronously growing microbial populations

Florian G. Pflug[1], Deepak Bhat[2], Simone Pigolotti[1*]

1 Biological Complexity Unit, Okinawa Institute of Science and Technology Graduate University, Onna, Okinawa 904-0495, Japan
2 Department of Physics, School of Advanced Sciences, Vellore Institute of Technology, Vellore, Tamil Nadu, India

* simone.pigolotti@oist.jp

Appendix A: Extensions of the theory

Non-exponential growth

Our approach can be generalized to populations that do not grow exponentially. We here assume that the number of genomes grows with time according to an arbitrary function $N_g(t)$. In this case, following the same logic of the Methods section, we find that Eq. (2) becomes

$$\mathcal{P}(x,t) = \int_0^\infty d\tau P(\tau;t) f(x,\tau) \tag{A1}$$

where $P(\tau;t) \propto N_g'(t-\tau)$. The proportionality constant can be determined by imposing normalization of $P(\tau)$. After integrating Eq. A1 by parts, we obtain

$$\mathcal{P}(x,t) = \langle N_g(t-\tau) \rangle \,. \tag{A2}$$

This generalization can be applied, for example, to bacterial or eukaryotic populations that grow in a partially synchronous manner. In such cases, the exponential growth $N_g(t)$ might be modulated by oscillations [1]. For bacteria such as *E. coli* grown using standard culture protocols, this effect is expected to be negligible, see Appendix 7 in [2].

We note that, in Eqs. (A1) and (A2) we implicitly made the assumption that the replication program $f(x,\tau)$ does not depend on t. The validity of this assumption should be verified for populations that are out of steady growth. Moreover, a practical limiting factor can be the accuracy at which $N_g(t)$ can be estimated.

Non-immortal genomes

For the purpose of computing the DNA abundance distribution, our assumption of immortal genomes is justified as long as typical genome lifetimes $\tau_{\max}$ are much longer than the doubling time $\tau_2 = \log(2)/\Lambda$. This is usually a realistic assumption, but in some cases one may want to generalize our approach to include genome degradation. In such case, the exponential distribution in Eq. (2) should be replaced with the age distribution $P(\tau)$ of surviving genomes. In particular, if genomes survive until age τ with probability $s(\tau)$, the age distribution is expressed by

$$P(\tau) \propto \Lambda e^{-\Lambda\tau} s(\tau) \tag{A3}$$

up to a normalization constant.

Multiple genome types

The theory we developed so far applies to statistically identical genomes. This assumption is sometimes referred to as "identity at birth" in the literature on renewal processes [3]: all genomes are born identical, and the differences that they might develop as they synthesize can be seen as independent outcomes of the same stochastic process.

There might be cases in which this assumption does not hold. For example, certain knockout mutations of *E. coli* lead to defective genome types that are inheritable [4]. To address these scenarios, we extend our theory to cases in which different genome types coexist in the population, each characterized by a different replication program.

We call $N_g^{(i)}(t)$ the number of genomes of type i at time t in the population, with $i = 1 \dots k$. The total number of genomes is $N_g(t) = \sum_i N_g^{(i)}(t)$. Each genome of type i can be replicated into a genome of type i' according a certain stochastic process. We assume that this stochastic process satisfies the Perron-Frobenius theorem, so that at long time the number of genomes are given by

$$N_g^{(i)}(t) \propto r_i \, e^{\Lambda t}. \tag{A4}$$

Here Λ is the leading eigenvalue of the dynamics, corresponding to the exponential growth rate of the population. The vector $(r_1 \dots r_k)$ is the leading eigenvector, having non-negative real entries according to the Perron-Frobenius theorem, and normalized such that $\sum_i r_i = 1$. The resulting replication program can then be obtained as an average over the genome types:

$$f(x, \tau) = \sum_{i=1}^{k} r_i f^{(i)}(x, \tau). \tag{A5}$$

This average replication program can be substituted into Eq. (2) to determine the DNA abundance distribution.

Numerical simulations and analogy with stochastic resetting

The models we considered in the Results section are analytically solvable. However, this might not be the case for more complex models. In this Appendix, we briefly present an interpretation of the DNA abundance distribution $\mathcal{P}(x)$ as the steady-state of a dynamical process implementing stochastic resetting [5, 6]. This analogy can be used for efficient numerical simulations.

The models introduced in Sections and are described by linear equations. Formally, we assume that we can always write the stochastic process associated with a certain DNA replication program in terms of linear evolution operator $\hat{\mathcal{L}}$, so that

$$\partial_\tau f(x, \tau) = \hat{\mathcal{L}} f(x, \tau). \tag{A6}$$

We now introduce a modified version of this equation:

$$\partial_\tau f(x, \tau) = \hat{\mathcal{L}} f(x, \tau) - \Lambda f(x, \tau). \tag{A7}$$

Equation A7 can be interpreted as describing a replication program equivalent to that of Eq. (A6), but that is stochastically reset to its beginning at rate Λ. Stochastic simulations of this process can be easily implemented numerically.

We note that $\mathcal{P}(x)$ is a steady solution of Eq. (A7), i.e., it satisfies

$$\left(\hat{\mathcal{L}} - \Lambda\right) \mathcal{P} = 0. \tag{A8}$$

This means that we can determine $\mathcal{P}(x)$, up to an appropriate normalization constant, by sampling the stochastic dynamics described Eq. (A7) at steady state. Such stochastic simulations can be efficiently implemented numerically [2].

Appendix B: Eukaryotic DNA replication

In this Appendix, we derive Eq. (9) for the DNA abundance in the case of origins at well-defined locations and with time-homogeneous firing rates. The general eukaryotic replication program is given by

$$f(x,\tau) = 1 - e^{-\int\int dx' d\tau' I(x',\tau')\theta\left(\tau-\tau'-\frac{|x-x'|}{v}\right)}, \tag{B1}$$

see [7,8]. Equation (B1) is equivalent to the expression $f(x,\tau) = 1 - \exp(-\iint_{V_{x,\tau}} dx'd\tau'\, I(x',\tau'))$ in the Main Text, but with an explicit expression for the past light cone. We note that

$$\frac{d}{d\tau}\int\int dx' d\tau' I(x',\tau')\theta\left(\tau-\tau'-\frac{|x-x'|}{v}\right) = \int dx' I\left(x', \tau - \frac{|x-x'|}{v}\right). \tag{B2}$$

We use Eq. (2) to obtain

$$\mathcal{P}(x) = \int_0^\infty dt \left[\int dx'' I\left(x'', \tau - \frac{|x-x''|}{v}\right)\right] \times \left[e^{-\Lambda\tau - \int\int dx' dt' I(x',\tau')\theta\left(\tau-\tau'-\frac{|x-x'|}{v}\right)}\right]. \tag{B3}$$

Setting $I(x,\tau) = \sum_{j=1}^K I_j(\tau)\delta(x-x_j)$, i.e. assuming discrete origins $x_1,\ldots,x_K$, yields

$$\mathcal{P}(x) = \int_0^\infty d\tau \left[\sum_j I_j\left(\tau - \frac{|x-x_j|}{v}\right)\right] \times \left[e^{-\Lambda\tau - \sum_j \int_0^{\tau-|x-x_j|/v} d\tau' I_j(\tau')}\right], \tag{B4}$$

and in the time homogeneous case $I_j(\tau) = I_j^*\theta(\tau)$ we find

$$\mathcal{P}(x) = 1 - \Lambda\int_0^\infty d\tau\, e^{-\Lambda\tau - \sum_j I_j^*\left(\tau-\frac{|x-x_j|}{v}\right)\theta\left(\tau-\frac{|x-x_j|}{v}\right)}. \tag{B5}$$

The integral can be then done by breaking it into different time intervals in which only a certain set of the theta functions are non-zero. This yields

$$\begin{aligned}&\int_0^\infty d\tau\, e^{-\Lambda\tau - \sum_j I_j^*(\tau-|x-x_j|/v)\theta(\tau-|x-x_j|/v)} \\ &= \frac{1-e^{-\Lambda\tau_1}}{\Lambda} \\ &+ \sum_{k=1}^{K-1} \frac{e^{-\Lambda\tau_k - \sum_{j=1}^k I_j^*(\tau_k-\tau_j)} - e^{-\Lambda\tau_{k+1} - \sum_{j=1}^k I_j^*(\tau_{k+1}-\tau_j)}}{\Lambda + \sum_{j=1}^k I_j^*} \\ &+ \frac{e^{-\Lambda\tau_K - \sum_{j=1}^K I_j^*(\tau_K-\tau_j)}}{\Lambda+\sum_{j=1}^K I_j^*}\end{aligned} \tag{B6}$$

where $\tau_k = \frac{|x-x_k|}{v}$ are the times it takes a replisome formed at the k-th origin to reach position x, and are ordered for a given x in such a way that $0 \le \tau_1 \le \cdots \le \tau_K$. Therefore, we have

$$\begin{aligned}\mathcal{P}(x) &= e^{-\Lambda\tau_1} \\ &- \Lambda\sum_{k=1}^{K-1} \frac{e^{-\Lambda\tau_k - \sum_{j=1}^k I_j^*(\tau_k-\tau_j)} - e^{-\Lambda t_{k+1} - \sum_{j=1}^k I_j^*(\tau_{k+1}-\tau_j)}}{\Lambda + \sum_{j=1}^k I_j^*} \\ &- \Lambda\frac{e^{-\Lambda\tau_K - \sum_{j=1}^K I_j^*(\tau_K-\tau_j)}}{\Lambda+\sum_{j=1}^K I_j^*}.\end{aligned} \tag{B7}$$

Equation (9) is obtained from Eq. (B7) by substituting $\mathcal{T}_k$ and W_k and grouping terms with the same exponent.

Appendix C: Eukaryotic origin inference by simulated annealing

We fit the solution of our eukaryotic model, Eq. (9), to experimentally measured DNA abundances in an exponentially growing asynchronous population of budding yest (*S. cerevisiae* W303) [9]. We correct for sequencing bias using the DNA abundance measured in a stationary population. Our input data $a_1, \ldots, a_N$ are thus the ratios of number of reads in the exponential vs. stationary population found within each 1kb window along the W303 genome (GenBank accession *CM007964.1*). To estimate number and positions of origins and their fork firing rates, we use simulated annealing [10].

Our free parameters are the number of origins K; their positions x_j and intensities I_j^*/v_0, with $j = 1 \ldots K$; and the ratio of growth rate to speed Λ/v_0. We collectively refer to the vector of $2K + 1$ free parameters (excluding K itself) as Θ. To prevent over fitting, we use the Akaike information criterion (AIC) as the cost function, defined as

$$\mathrm{AIC}(\Theta) = 2(2\mathrm{K} + 1) - 2 \log \mathrm{L}(\Theta). \tag{C1}$$

The first term on the right hand side of Eq. (C1) penalizes a large number of origins, while the second term penalizes small log-likelihoods. The quantity $\log L(\Theta)$ is computed as in Eq. (H2). The noise in the data appears larger than expected by Poissonian sampling. We therefore use the empirical noise estimate $\sigma_i = \sigma = \sqrt{\frac{1}{N-1} \sum_i (a_{i+1} - a_i)^2/2} \approx 3.3 \cdot 10^{-9}$ instead of the Poissonian error estimates from Eq. (H1). Our estimate is based on the assumption that the systematic contribution to the quantity σ is negligible compared to the noise. We confirmed post-hoc this assumption by replacing the a_i with the fitted model predictions. The resulting value $2 \cdot 10^{-10}$ is an order of magnitude smaller; σ therefore indeed mainly represents measurement errors rather than systematic effects.

Our simulated annealing algorithm comprises the following steps:

1. We set the Monte-Carlo temperature to $T_m = 200$ and generate an initial parameter vector Θ. We draw Λ/v_0 uniformly from $[7.7 \times 10^{-8}, 2.3 \times 10^{-5}]$ bp^{-1} and place two origins randomly on each chromosomes with intensities $I_j^\star/v_0$ drawn uniformly from $[1.1 \times 10^{-7}, 1.6 \times 10^{-3}]$ bp^{-1}.

2. We generate a parameter proposal Θ' by executing one of the following moves with the indicated probability:

   - *Relocating an origin (prob. 24%).* We choose one among the K origins randomly and relocate it to a uniformly drawn position on the same chromosome. The origin firing rate remains unchanged.
   - *Altering one origin intensity (prob. 24%).* We randomly choose one of the origins and replace its intensity rate $I_j^\star/v_0$ with a value drawn uniformly from $[1.1 \times 10^{-7}, 1.6 \times 10^{-3}]$ bp^{-1}.
   - *Removing an origin (prob. 24%).* We choose one among the K origins and remove it unless it is the only origin on the chromosome.
   - *Adding an origin (prob. 24%).* We add an origin at a uniformly drawn location on the genome with an intensity uniformly drawn from $[1.1 \times 10^{-7}, 1.6 \times 10^{-3}]$ bp^{-1}.
   - *Altering the growth rate (prob. 4%).* We draw a new value of Λ/v uniformly from $[7.7 \times 10^{-8}, 2.3 \times 10^{-5}]$ bp^{-1}.

The ranges of free parameters are chosen to cover replisome speeds (v) from 10bps^{-1} to 100bps^{-1}, doubling times $\ln 2/\Lambda$ between 50 minutes to 150 minutes, and fork firing delays $1/I_j^*$ between 1 minute and 150 minutes.

3. We accept Θ' with Metropolis probability

$$q = \min\left\{1,\ \exp\left(\frac{\text{AIC}(\Theta) - \text{AIC}(\Theta')}{T_m}\right)\right\}. \tag{C2}$$

4. We reduce the Monte-Carlo temperature by a factor $1 - 1.336 \cdot 10^{-5}$ and reiterate from step (2). The total number of iterations is $2 \cdot 10^6$. With this choice, the final temperature is equal to $T_m = 5 \cdot 10^{-10}$.

After half the number of iterations (10^6), the number of origins is already close to the final value (222 vs. the final number of 234), and repeating the procedure 10 times yield similar final numbers of origins (225 ± 6). We take this as evidence that the algorithm has converged after $2 \cdot 10^6$ iterations.

Appendix D: Bacterial DNA replication with time-dependent speed

We consider the Langevin Eqs. (11). We write the associated Fokker-Planck equation for the first replisome:

$$\frac{\partial}{\partial \tau} p = h(\tau)\left(-v_0 \frac{\partial}{\partial y} p + D \frac{\partial^2}{\partial y^2} p\right). \tag{D1}$$

Equation (D1) shows that the function $h(\tau)$ acts as global time rescaling. Therefore, if $\hat{p}$ is a solution of the Fokker-Planck equation with constant drift and diffusion terms

$$\frac{\partial}{\partial \tau}\hat{p} = -v_0 \frac{\partial}{\partial y}\hat{p} + D \frac{\partial^2}{\partial y^2}\hat{p} \tag{D2}$$

under certain boundary and initial conditions, then

$$p(y,\tau) = \hat{p}\big(y, H(\tau)\big) \tag{D3}$$

solves the original Fokker-Planck Eq. (D1) with the same boundary and initial conditions, where $H(\tau) = \int_0^\tau du\, h(u)$. We impose, in particular, an absorbing boundary condition at x, $\hat{p}(x,\tau) = 0\ \forall \tau$ and an initial condition $p(y, \tau = 0) = \delta(y)$. The flux $\hat{J} = v_0 p - D\partial_y p$ through the absorbing boundary is given by an inverse Gaussian distribution:

$$\hat{J}(x,\tau) = \frac{x}{\sqrt{4\pi D \tau^3}} e^{-\frac{(x - v_0\tau)^2}{4D\tau}}. \tag{D4}$$

Since the first-passage density of Eq. (D1) equals the flux $J(x,\tau) = h(\tau)\hat{J}\big(x, H(\tau)\big)$, we have

$$\frac{\partial}{\partial \tau} f_1(x,\tau) = h(\tau)\hat{J}\big(x, H(\tau)\big) = \frac{h(\tau)x}{\sqrt{4\pi D H^3(\tau)}} e^{-\frac{(x - v_0 H(\tau))^2}{4DH(\tau)}}. \tag{D5}$$

The same approach can be used for the second replisome after a suitable change of coordinates and initial conditions. Integrating Eq. (D5) over τ, and similarly for the second replisome, directly leads to Eqs. (12) and (13).

Appendix E: Effect of diffusivity on the shape of the bacterial DNA abundance distribution

We here study the effect of increasing the diffusivity D on the shape of the DNA abundance distribution $\mathcal{P}(x)$. We first focus on the the meeting point region, and then consider the region far from the expected meeting point. We call the expected meeting point $x = L/2$ the "replication terminus". Since this is the last location on the genome to be replicated, $\mathcal{P}$ attains its global minimum there. For $D = 0$, $\mathcal{P}$ exhibits a cusp at the terminus, and thus infinite curvature. For $D > 0$, the cusp vanishes, the curvature is finite and decreases with D (Fig. 4c-d).

To quantitatively link D with this curvature, we first use that Eq. (11) is symmetric under the swap of replisomes and mirroring of the genomic coordinate: $f_1(x,\tau) = f_2(L-x,\tau)$. As a consequence, $f'(L/2)$ vanishes for all time τ and therefore $\mathcal{P}'(L/2) = 0$ according to Eq. 2. The curvature at $x = L/2$ is thus simply $\mathcal{P}''(L/2)$. We focus on the relative curvature expressed by

$$\frac{\mathcal{P}''(L/2)}{\mathcal{P}(L/2)} = \frac{\int_0^\infty d\tau \Lambda e^{-\Lambda\tau} f''(L/2,\tau)}{\int_0^\infty d\tau \Lambda e^{-\Lambda\tau} f(L/2,\tau)}. \tag{E1}$$

To make progress, we approximate the replication time distribution with a Gaussian:

$$\psi(x,\tau) = \frac{\partial}{\partial\tau} f(x,\tau) \approx \frac{1}{\sqrt{2\pi\sigma^2}} e^{-\frac{(t-\mu(x))^2}{2\sigma^2(x)}}, \tag{E2}$$

where the mean $\mu(x)$ and the variance $\sigma^2(x)$ have to be determined. It then follows from Eq. (3) that

$$\mathcal{P}(x) = \int_0^\infty d\tau e^{-\Lambda\tau}\psi(x,\tau) \approx e^{-\mu(x)\Lambda + \sigma^2(x)\Lambda^2/2}. \tag{E3}$$

Here, we have approximated the integral by extending the lower extreme to $-\infty$. We expect the error caused by this approximation to be negligible, since the Gaussian distribution that approximates $\psi(x,\tau)$ must be concentrated on the positive real numbers. By using that $f'(L/2,\tau) \equiv 0$ and thus $\mu'(x) = 0$, and assuming that the dependence of σ on x is small enough to be ignored, we obtain

$$\frac{\mathcal{P}''(L/2)}{\mathcal{P}(L/2)} \approx -\Lambda\mu''(L/2). \tag{E4}$$

We now have to evaluate $\mu''(L/2) = \partial_x^2 \int \tau\partial_\tau f(x,\tau) d\tau|_{x=L/2}$. To make this tractable, we now further approximate the individual inverse Gaussian laws of f_1, f_2 from Eq. (12) with Gaussian distributions. For these individual laws, the means and variances are given by Eq. (13) which yields

$$\hat{f}(x,\tau) = 1 - \left[1 - \Phi\left(\frac{\tau - x/v_0}{\sqrt{2Dx/v_0^3}}\right)\right] \times \left[1 - \Phi\left(\frac{\tau - (L-x)/v_0}{\sqrt{2D(L-x)/v_0^3}}\right)\right]. \tag{E5}$$

where we have for simplicity assumed that the replisome speed is constant, i.e. $h(\tau) = 1$.

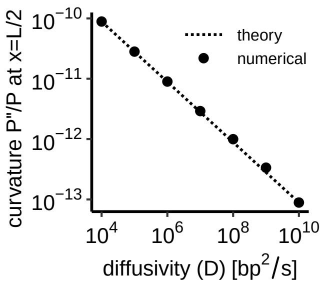

Fig E1. Curvature $\mathcal{P}''(x)/\mathcal{P}(x)$ at the terminus $x = L/2$ predicted by Eq. (E7) vs. numerical simulations.

Therefore:

$$\begin{aligned}
\mu''(L/2) &\approx \frac{\partial^2}{\partial x^2} \int_{-\infty}^{\infty} \tau \frac{\partial}{\partial \tau} \hat{f}(x,\tau) d\tau \Big|_{x=L/2} \\
&= \frac{\partial^2}{\partial x^2} \left(\int_0^{\infty} d\tau \big(1 - \hat{f}(x,\tau)\big) - \int_{-\infty}^{0} d\tau \hat{f}(x,\tau) \right) \Big|_{x=L/2} \\
&= - \int_{-\infty}^{\infty} \hat{f}''(L/2,\tau) d\tau \\
&= - \frac{2}{\sqrt{\pi D L v_0}}
\end{aligned} \tag{E6}$$

and by inserting into Eq. (E4) we obtain the normalized curvature

$$\frac{\mathcal{P}''(L/2)}{\mathcal{P}(L/2)} \approx \frac{2\Lambda}{\sqrt{\pi D L v_0}}. \tag{E7}$$

The curvature predicted by Eq. (E7) agrees very well with numerical estimates sespite the various approximations made in the derivation (Fig. E1).

Eq. E3 also permits to approximate the value of $\mathcal{P}(x)$ itself at the terminus. We use that

$$\begin{aligned}
\mu(L/2) &\approx \int_{-\infty}^{\infty} \tau \frac{\partial}{\partial \tau} \hat{f}(x,\tau) d\tau \\
&= \int_0^{\infty} \big(1 - \hat{f}(x,\tau)\big) d\tau - \int_{-\infty}^{0} \hat{f}(x,\tau) d\tau \\
&= \frac{L}{2v_0} - \sqrt{\frac{DL}{\pi v_0^3}}
\end{aligned} \tag{E8}$$

and

$$\begin{aligned}
\sigma^2(L/2) &\approx \int_{-\infty}^{\infty} \tau^2 \frac{\partial}{\partial \tau} \hat{f}(x,\tau) d\tau - \mu^2(L/2) \\
&= 2 \int_0^{\infty} \tau \big(1 - \hat{f}(x,\tau)\big) d\tau \\
&\quad - 2 \int_{-\infty}^{0} \tau \hat{f}(x,\tau) d\tau - \mu^2(L/2) \\
&= \frac{\pi - 1}{\pi} \frac{DL}{v_0^3}.
\end{aligned} \tag{E9}$$

Approximating $\mu(L/2+\epsilon) \approx \mu(L/2) + \epsilon^2 \mu''(L/2)/2$ and $\sigma^2(L/2+\epsilon) \approx \sigma^2(L/2)$ in Eq. (E3) yields

$$\mathcal{P}(L/2+\epsilon) \approx \exp\left[-\frac{\Lambda L}{2v_0} + \Lambda\sqrt{\frac{DL}{\pi v_0^3}} + \frac{\pi-1}{\pi}\frac{\Lambda^2 DL}{2v_0^3} + \epsilon^2 \frac{\Lambda}{\sqrt{\pi DL v_0}}\right] \tag{E10}$$

close to the replication terminus. The curvature at $x = L/2$ is therefore

$$\mathcal{P}''(L/2) \approx \frac{2\Lambda}{\sqrt{\pi DL v_0}} \exp\left[-\frac{\Lambda L}{2v_0} + \Lambda\sqrt{\frac{DL}{\pi v_0^3}} + \frac{\pi-1}{\pi}\frac{\Lambda^2 DL}{2v_0^3}\right]. \tag{E11}$$

We now study the shape of the DNA abundance far from the terminus region. Within these regions, only one of the two replisomes plays a relevant role. For simplicity, we focus on the region replicated by replisome 1. Also in this case, we assume constant replisome speed, i.e. $h(\tau) = 1$. Since replisome 2 has virtually no chance of reaching the area of interest before replisome 1, we assume $f(x,\tau) \approx f_1(x,\tau)$. The DNA abundance $\mathcal{P}(x)$ can then be explicitly found from the characteristic function of the inverse Gaussian distribution; the expansion $\sqrt{1+x} \approx 1 + x/2 - x^2/8$ yields for small D

$$\mathcal{P}(x) \approx \exp\left[\left(-\frac{\Lambda}{v_0} + D\frac{\Lambda^2}{v_0^3}\right)x\right]. \tag{E12}$$

The change in the exponential decay rate due to D is therefore of order $D\Lambda^2/v^3$.

Appendix F: Uncertainty on the meeting point

We now consider the random point Z at which the two replisomes meet and its uncertainty $\langle (Z - \langle Z\rangle)^2\rangle$. We call $\tau_1(x)$ and $\tau_2(x)$ the random times at which replisomes 1 and 2 reach location x, respectively. Then, $\tau_1(z) < \tau_2(z)$ for all points z to the left of the meeting point Z, and $\tau_1(z) > \tau_2(z)$ for all points to the right of Z. Therefore

$$\mathbb{P}(Z < z) = \mathbb{P}(\tau_1(z) > \tau_2(z)) \,. \tag{F1}$$

We note that the means and variances in Eq. (13) are the means and variances of $H\big(\tau_i(x)\big)$. Since $H(t)$ increases monotonically, we substitute it into Eq. (F1):

$$\mathbb{P}(Z < z) = \mathbb{P}\big(H(\tau_2(z)) - H(\tau_1(z)) < 0\big). \tag{F2}$$

By using that $\tau_1(z)$ and $\tau_2(z)$ are independent, and substituting the means and variances from Eq. (13), we find the mean and variance of $H(\tau_2(z)) - H(\tau_1(z))$ to be $(L-2z)/v_0$ and $2DL/v_0^3$, respectively. By approximating the distribution of the difference between the two replication times with a Gaussian, we obtain

$$\mathbb{P}(Z < z) \approx \Phi\left(\frac{-(L-2z)/v_0}{\sqrt{2DL/v_0^3}}\right) = \Phi\left(\frac{z - L/2}{\sqrt{DL/2v_0}}\right) \tag{F3}$$

where $\Phi(x) = 1/2 + \mathrm{erf}\left(x/\sqrt{2}\right)/2$ is the cumulative function of the Gaussian distribution. The uncertainty about the meeting point is therefore given by Eq. (14). Despite the Gaussian approximation used in the derivation of Eq. (14), the predicted uncertainty agrees with numerical estimates across a wide range of diffusivities D (Fig. F1).

In E. coli, the maximum possible uncertainty of the meeting point is restricted by the Tus-*Ter* System. This system comprises oriented DNA motifs, so-called *Ter* sites,

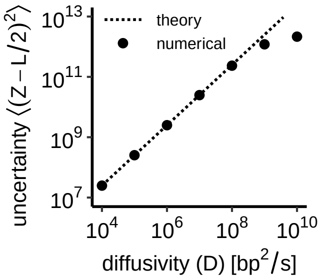

Fig F1. Uncertainty of the meeting point Z predicted by Eq. (14) vs. numerical simulations.

that only allow replisomes to pass in a certain direction when bound to a Tus protein. *Ter* sites are arranged around the expected termination point (180° from the origin) in such a way that replisomes which have already passed the expected termination point are stopped at the next *Ter* they encounter [11, 12].

The two relevant *Ter* sites are *terC* which stops forward-moving replisomes 4kb after the expected meeting point, and *terA* which stops backward-moving replisomes 263kb after the expected meeting point. For diffusivity $D \approx 0$, the Tus-*Ter* system thus shifts the replisome meeting point by 4kb which lies below the spatial resolution of the marker-frequency analysis data for E. coli (10kb). Simulations show that the effect of the Tus-*Ter* system is similarly negligible for the values of D from Table 1. When the diffusivity increased 10×, the effect of the Tus-*Ter* system becomes noticable, and when increased 100×, the effect becomes pronounced, see Fig. F2.

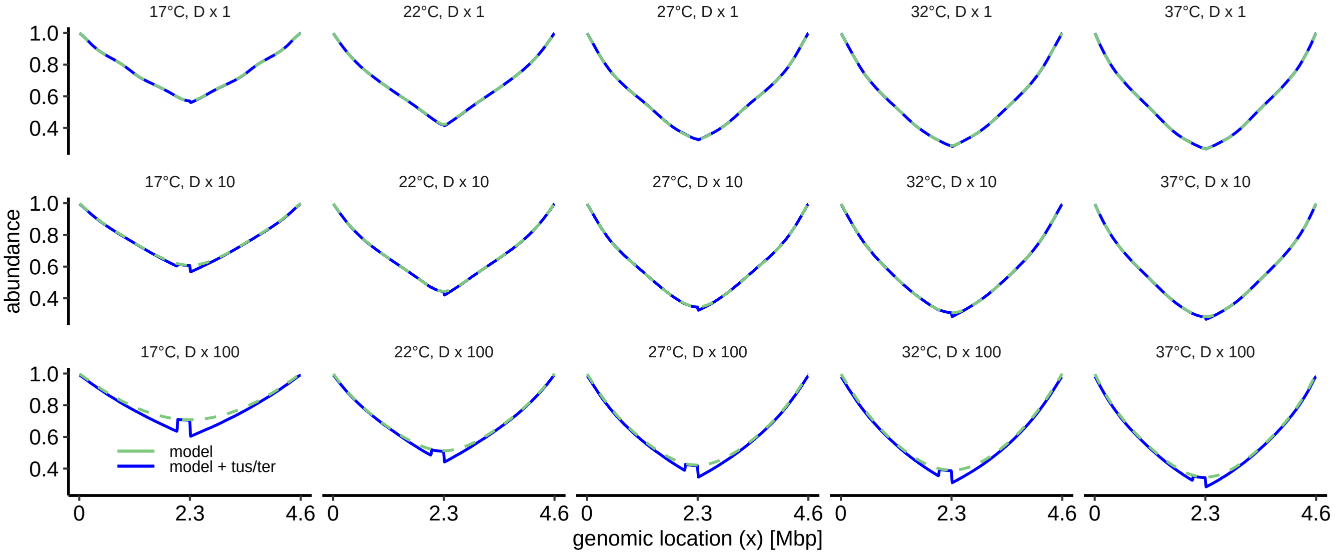

Fig F2. Effect of the Tus-*Ter* system. Plot show our unmodified model (green) and simulated abundances for a model which includes the Tus-*Ter* system (blue). In the Tus-*Ter* model, the forward-moving replisome arrests when it reaches *terC* (4kb after the expected meeting point) and the backward-moving replisome arrests at *terA* (263kb after the expected meeting point) [11, 12]. For simplicity, we assume perfectly efficient blocking of replisomes by Tus-bound *Ter* sites. In reality, blocking efficiency is finite [13], so that we expect the effect of these sequences on the DNA anundance to be even weaker. Abundances are based on the estimated model parameters per Table 1 (first row) as well as parameters with a 10-fold (second row) and 100-fold (third row) increase in diffusivity.

Appendix G: Bacterial DNA replication with position-dependent speed

In this Appendix, we solve a model with position-dependent speeds $v_1(x)$ and $v_2(x)$ in the limit of small diffusivity. This will allow us to directly compare time-dependent and position-dependent speed models. We allow the diffusion constants $D_1(x)$, $D_2(x)$ to vary in space as well. The Langevin equations are:

$$\frac{d}{d\tau}x_1 = v_1(x) + \sqrt{2D_1(x)}\,\xi_1(\tau)$$
$$\frac{d}{d\tau}x_2 = v_2(x) + \sqrt{2D_2(x)}\,\xi_2(\tau). \tag{G1}$$

where $v_1(x) > 0$ and $v_2(x) < 0$. As in the case of Eq. (11), the initial conditions are $x_1(0) = 0$, $x_2(0) = L$ and we define replication programs $f_1(x,\tau)$, $f_2(x,\tau)$ via the first-passage times of $x_1(\tau)$ and $x_2(\tau)$, respectively, through position x.

We now assume that (i) $v_1(x)$, $v_2(x)$ change only slowly, (ii) $D_1(x)$, $D_2(x)$ are small in comparison, and (iii) x is not too close to the replication origin. Under these assumptions, replication times are approximately additive, meaning that if $\tau_{x_0\to x_1}$ is the (random) time a replisome originating from x_0 takes to reach x_1, then $\tau_{x_0\to x_1}$ and $\tau_{x_1\to x_2}$ are independent, and $\tau_{x_0\to x_2} = \tau_{x_0\to x_1} + \tau_{x_1\to x_2}$. For large enough x, we invoke the central limit theorem for the sum $\tau_x = \tau_{x_0\to x_1} + \cdots + \tau_{x_k\to x}$ and conclude that ψ_1 and ψ_2 are approximately Gaussian, implying

$$f_i(x,\tau) \approx \frac{1}{2} + \frac{1}{2}\,\mathrm{erf}\left(\frac{\tau - \mu_i(x)}{\sqrt{2}\sigma_i(x)}\right) \tag{G2}$$

To find the position-dependent mean $\mu_i(x)$ and variance $\sigma_i^2(x)$, we rely on $v_i(x)$ changing sufficiently slowly relative to $D_i(x)$ so that we can find a subdivision $x_0 \leq \cdots \leq x_k \leq x$ where $v_i(x)$ is approximately constant in each interval. By approximating the mean and variance of $\tau_{x_l\to x_{l+1}}$ with the constant-speed solution, we obtain

$$\mu_i(x) = \int_{x_i(0)}^{x} \frac{dx'}{v_i(x')}, \quad \sigma_i^2(x) = \int_{x_i(0)}^{x} \frac{2D_i(x')}{v_i^3(x')}dx'. \tag{G3}$$

We now compute μ_i and σ_i^2 for the position-dependent speed fluctuations from Ref. [2] of the form

$$v(x) = \overline{v}\,(1 + \delta\cos(\omega x + \phi)) \tag{G4}$$

where x is the distance of the replisome from the origin. We note that because we express $v(x)$ in terms of distance travelled, the speed of the two replisomes when they traverse the same physical location will in general differ. Since integration of $1/v(x)$ and $1/v^3(x)$ is cumbersome, we first compute the following integrals of powers of $g(u) = 1 + \delta\cos u$, which are valid for $z \in (-\pi, \pi)$:

$$\hat{G}_1(z) = \int_0^z \frac{du}{g(u)} = \frac{2\tanh^{-1}\left(\frac{(1-\delta)\tan\frac{z}{2}}{\sqrt{1-\delta^2}}\right)}{\sqrt{1-\delta^2}}$$
$$\hat{G}_3(z) = \int_0^z \frac{du}{g^3(u)} = \frac{\left(\delta^2+2\right)\tanh^{-1}\left(\frac{(1-\delta)\tan\frac{z}{2}}{\sqrt{1-\delta^2}}\right)}{\left(1-\delta^2\right)^{5/2}} + \frac{\delta\sin(z)\left(\delta^2 - 3\delta\cos(z) - 4\right)}{2\left(1-\delta^2\right)^2(\delta\cos(z)+1)^2} \tag{G5}$$

T	$\overline{v}$ [bp/s]	D [kbp^2/s]	δ	ω [rad/Mbp]	ϕ [rad]
17	230 ± 22	1.0 ± 1.2	0.18 ± 0.07	6.2 ± 6.7	2.3 ± 1.6
22	350 ± 37	1.0 ± 1.6	0.23 ± 0.15	2.8 ± 0.73	3.4 ± 0.72
27	540 ± 17	0.4 ± 0.5	0.18 ± 0.04	4.6 ± 0.11	2.1 ± 0.11
32	820 ± 57	1.4 ± 2.3	0.12 ± 0.04	5.5 ± 0.13	1.6 ± 0.16
37	970 ± 25	2.9 ± 2.3	0.18 ± 0.02	4.3 ± 0.15	3.0 ± 0.14

Table G1. Parameter estimates for position-dependent speed $v(x) = \overline{v}\big(1 + \delta\cos(\omega x + \phi)\big)$ model of Bhat *et al.* [2].

We also introduce the definite integrals

$$\begin{aligned}\overline{G}_1 &= \int_{-\pi}^{\pi} \frac{du}{g(u)} = \frac{2\pi}{\sqrt{1-\delta^2}},\\ \overline{G}_3 &= \int_{-\pi}^{\pi} \frac{dz}{g^3(z)} = \frac{\pi\left(\delta^2+2\right)}{\left(1-\delta^2\right)^{5/2}}.\end{aligned} \tag{G6}$$

Writing $[[\cdot]]$ for the nearest integer we obtain

$$G_p(z) = \int_0^z \frac{du}{g^p(u)} = \hat{G}_p(z) + \overline{G}_p \left[\left[\frac{z}{2\pi}\right]\right] \tag{G7}$$

and in terms of G_p we finally have

$$V_p(x) = \int_0^x \frac{du}{v^p(u)} = \frac{v_0}{\omega}\big(G_p(\omega x + \phi) - G_p(\phi)\big). \tag{G8}$$

By setting $v_1(x) = v(x)$, $v_2(x) = -v(L-x)$ and $D_1(t) = D_2(t) = D$ in Eq. (G3) and inserting the means and variances into Eq. (G2), we arrive at the replication program

$$f(x,\tau) = 1 - \frac{1}{4}\,\mathrm{erfc}\left(\frac{t - V_1(x)}{2^{\frac{3}{2}} D V_3(x)}\right)\mathrm{erfc}\left(\frac{t - V_1(L-x)}{2^{\frac{3}{2}} D V_3(L-x)}\right). \tag{G9}$$

Comparison of parameter estimates

Most parameter estimates for the position-dependent speed model obtained using the procedure outlined in S1 Appendix H agree well with the values reported in Ref. [2], see Fig. G1a. Estimates that deviate also exhibit large uncertainties between replicates, see Table G1. These large uncertainties can be explained in a similar way as for the time-dependent model presented in Section .

Parameter estimates also agree well between time- and position-modulated models, see Fig.G1b. The oscillation frequency ω is not directly comparable between the two models, but when plotted against replication progress both models yield similar oscillations, see Fig. G2a. When the two models are compared across samples, we find that, for a large majority of samples, the time-dependent speed model yields a slightly higher likelihood than the position-dependent speed model, see Fig. G2b.

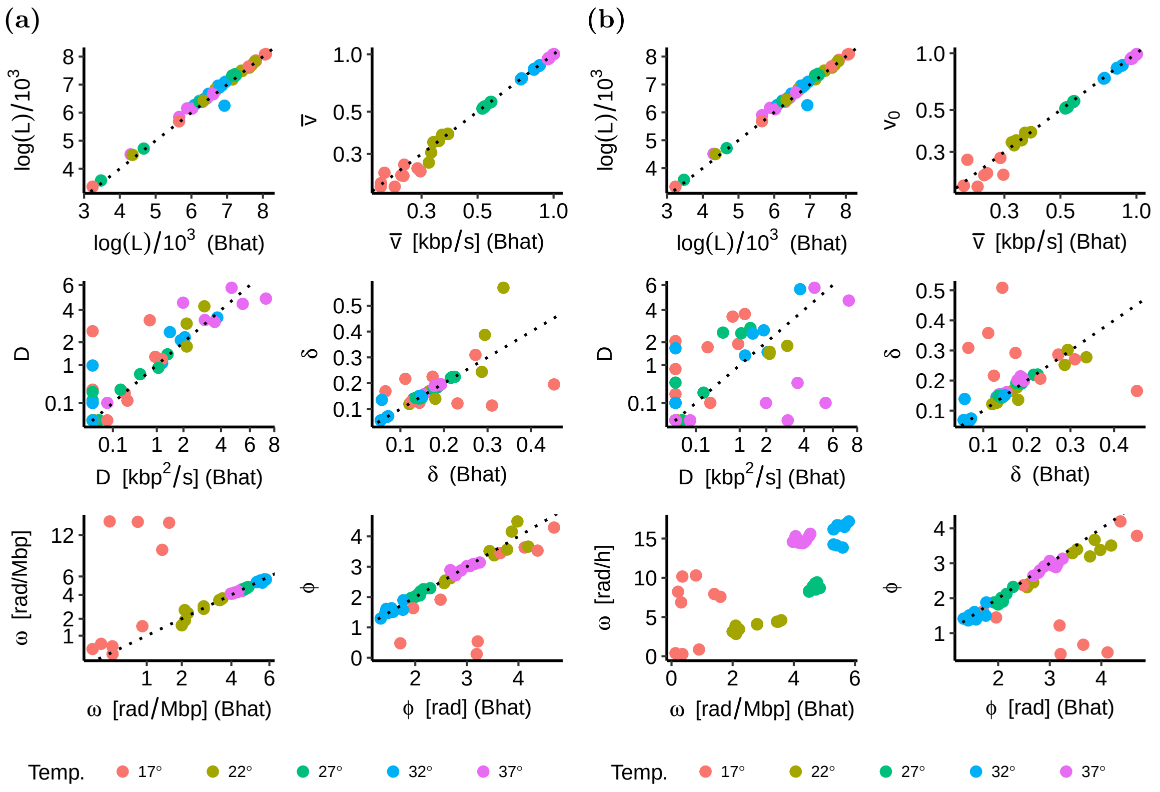

Fig G1. Comparison of our parameter estimates with those of Ref. [2]. The plots shows data points for all 9 combinations of biological replicate (3 per temperature) and stationary reference sample (3 in total, taken at temperature 17°C, 27°C and 37°C). **(a)**. Position-dependent speed $v(x) = \overline{v}\big(1 + \delta \cos(\omega x + \phi)\big)$. **(b)**. Time-dependent speed $v(\tau) = v_0\big(1 + \delta \cos(\omega\tau + \phi)\big)$.

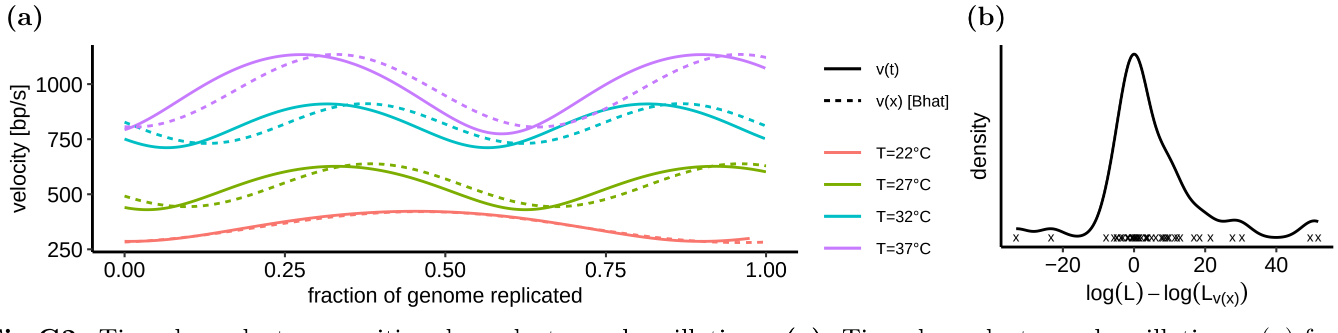

Fig G2. Time-dependent vs. position-dependent speed oscillations. **(a)**. Time-dependent speed oscillations $v(\tau)$ from figure 5c vs. the position-dependent speed oscillations from Bhat *et al.* [2]. **(b)**. Likelihoods L of the best-fitting time-dependent speed model vs. $L_{v(x)}$ of the best-fitting position-dependent model (computed as described in S1 Appendix H).

Appendix H: Parameter estimation for bacterial models

We compute the position-wise bias-corrected abundances and their uncertainties as

$$a_i = \frac{n_i^{\mathrm{exp}}}{n_i^{\mathrm{stat}}}, \qquad \sigma_i = a_i \sqrt{\frac{1}{n_i^{\mathrm{exp}}} + \frac{1}{n_i^{\mathrm{stat}}}} \tag{H1}$$

given the experimentally observed read counts at genomic locations $x_1, \ldots, x_n$ in an exponentially growing (n_i^{exp}) and stationary (n_i^{stat}) populations [2]. Since the counts n_i^{exp} and n_i^{stat} are typically large, we approximate the error distributions as Gaussian. Defining the parameter vector $\Theta = \big(v_0, D, \delta, \omega, \phi\big)$, we express the log likelihood by

$$\begin{aligned} \log L(\Theta) = &-\frac{N}{2}\log(2\pi) - \sum_{i=1}^{N} \log(\sigma_i) \\ &- \frac{1}{2}\sum_{i=1}^{N} \frac{\big(a_i - \lambda \mathcal{P}(x_i|\Theta)\big)^2}{\sigma_i^2}. \end{aligned} \tag{H2}$$

where $\lambda = \sum_i a_i \big/ \sum_i \mathcal{P}(x_i|\Theta)$ is a scaling factor.

We evaluate $\mathcal{P}(x|\Theta)$ by a numerical integration of Eq.(3). This procedure requires a limited computational effort compared with Ref. [2], that evaluated the likelihood using stochastic simulations. We fit two different models corresponding to two different replication programs with are both instances of the general bacterial program from Eq. (10): (1) the time-dependent speed model in Eq. (12) with the modulating function $h(\tau)$ given in Eq.(15). (2) the position-dependent speed model of Ref. [2] with replication program Eq. (G9), see S1 Appendix G (parameters are $\Theta = \big(\overline{v}, D, \delta, \omega, \phi\big)$ in this case, the parameter $\overline{v}$ takes the place of v_0).

The likelihood surfaces of these models are quite rough, in particular with respect to ω and ϕ. This causes combined gradient-ascent optimization of all five parameters to remain stuck in local maxima, unless the initial estimate is close to the global optimum. On the other hand, exhaustive exploration of the parameter space for all 5 parameters at sufficient resolution is computationally expensive. We thus used the following procedure:

1. We fit the *constant-speed, no-diffusion* ($\delta = 0$, $D = 0$) regime by optimizing v_0 while keeping $D = 0$ and $\delta = 0$. Initial value is $v_0 = 500$bp/s.

2. We fit the *oscillatory no-diffusion* ($\delta, \omega, \phi \geq 0$, $D = 0$) regime by optimizing v_0, δ, ω and ϕ while keeping $D = 0$. Initial values lie on a grid formed by the constant-speed, no-diffusion estimate of v_0, $\delta \in \{0, 1/2\}$, 25 logarithmically spaced values for ω from $\pi L/2$ to $32\pi L$ (L being the genome length), and 5 uniformly spaced values for ϕ from 0 to 2π.

3. We fit the *constant-speed diffusive* ($\delta = 0$, $D \geq 0$) regime by optimizing v_0 and D while keeping $\delta = 0$. Initial values lie on a grid formed by the constant-speed, no-diffusion estimate of v_0, and 21 logarithmically spaced values for D from 10 to 10^{10}.

4. We find the initial parameters for the *oscillatory diffusive* ($\delta, \omega, \phi \geq 0$, $D \geq 0$) regime by optimizing v_0, δ, ω and ϕ while fixing D to the *constant-speed diffusive* estimate. Initial values are the same as in (2), except that for v_0 we now use the *constant-speed diffusive* estimate.

5. We fit the *oscillatory diffusive* regime by optimizing v_0, D, δ, ω and ϕ, using the estimates found in (1)-(4) as initial values.

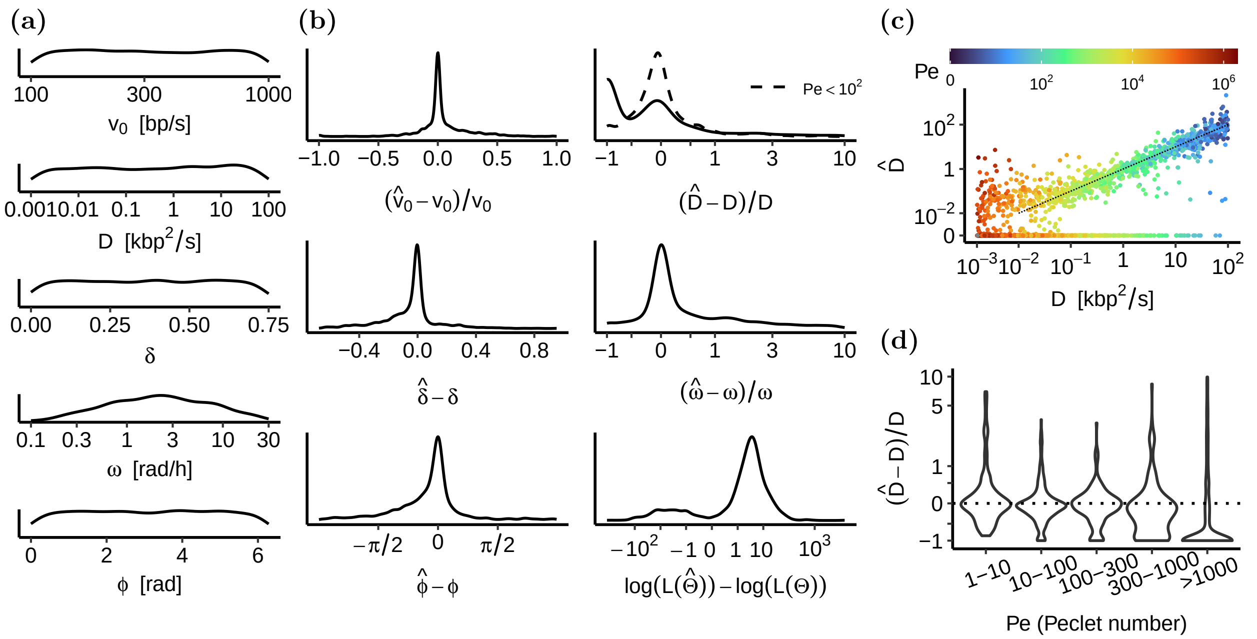

Fig H1. Parameter recovery. **(a)**. Distributions of true parameters θ. **(b)**. Estimation errors for simulated abundance profiles with $\Lambda = 1\,h^{-1}$ and 40k reads / 10kbp window on average. **(c)**. True value D vs. recovered value $\hat{D}$ for different ranges of the Peclet number $\mathrm{Pe} = Lv_0/4D$. **(d)**. Distribution of the relative error of $\hat{D}$ for different ranges of Pe.

6. We select the parameter regime with the lowest AIC score.

Identifiability

To study the identifiability of parameters v_0, D, δ, ω, ϕ of the bacterial replication program Eq. 12 with $h(t) = v_0\big(1 + \cos(\omega t + \phi)\big)$ we generated artificial DNA abundance distributions and estimated the parameters values using the procedure outlined in Appendix H. We randomly selected 2000 sets of parameters (Fig. H1a), and computed their predicted abundance profiles by simulating 10^6 genomes using a fixed growth rate of $\Lambda = 1\mathrm{h}^{-1}$). We then generated Poisson-distributed read counts n_i^{exp} (and similarly for n_i^{stat}, using a flat abundance profile) from these abundance profiles, and ran the estimation procedure described in S1 Appendix H. All parameters except D were recovered well (Fig. H1b).

Diffusivities D below a certain value are unlikely to pass the Akaike information criterion and are therefore estimated to be zero. This occurs frequently for parameter sets corresponding to a Peclet number $\mathrm{Pe} = Lv_0/4D$ larger than 300 (Fig. H1c-d).

References

1. Jafarpour F. Cell size regulation induces sustained oscillations in the population growth rate. Physical Review Letters. 2019;122(11):118101.

2. Bhat D, Hauf S, Plessy C, Yokobayashi Y, Pigolotti S. Speed variations of bacterial replisomes. Elife. 2022;11:e75884.

3. Boldin B, Diekmann O, Metz J. Population growth in discrete time: a renewal equation oriented survey. arXiv preprint arXiv:230609193. 2023;.

4. Sinha AK, Possoz C, Durand A, Desfontaines JM, Barre FX, Leach DR, et al. Broken replication forks trigger heritable DNA breaks in the terminus of a circular chromosome. PLoS genetics. 2018;14(3):e1007256.

5. Evans MR, Majumdar SN. Diffusion with stochastic resetting. Physical review letters. 2011;106(16):160601.

6. Evans MR, Majumdar SN, Schehr G. Stochastic resetting and applications. Journal of Physics A: Mathematical and Theoretical. 2020;53(19):193001.

7. Bechhoefer J, Rhind N. Replication timing and its emergence from stochastic processes. Trends in Genetics. 2012;28(8):374–381.

8. Baker A, Bechhoefer J. Inferring the spatiotemporal DNA replication program from noisy data. Physical Review E. 2014;89(3):032703.

9. Müller CA, Hawkins M, Retkute R, Malla S, Wilson R, Blythe MJ, et al. The dynamics of genome replication using deep sequencing. Nucleic Acids Research. 2013;42(1):e3–e3. doi:10.1093/nar/gkt878.

10. Bertsimas D, Tsitsiklis J. Simulated Annealing. Statistical Science. 1993;8(1):10 – 15. doi:10.1214/ss/1177011077.

11. Hill TM. Arrest of bacterial DNA replication. Annual Review of Microbiology. 1992;46:603–633.

12. Neylon C, Kralicek AV, Hill TM, Dixon NE. Replication Termination in Escherichia coli: Structure and Antihelicase Activity of the Tus-Ter Complex. Microbiology and Molecular Biology Reviews. 2005;69(3):501–526.

13. Elshenawy MM, Jergic S, Xu ZQ, Sobhy MA, Takahashi M, Oakley AJ, et al. Replisome speed determines the efficiency of the Tus- Ter replication termination barrier. Nature. 2015;525(7569):394–398.
