# Supplementary material for: Genome replication in asynchronously growing microbial populations
Source: PLoS Comput Biol. 2024 Jan 5;20(1):e1011753. doi: 10.1371/journal.pcbi.1011753 (PMC10796026; doi:10.1371/journal.pcbi.1011753)
Supplement: Attachment — Submitted filename: Reviewers_response.pdf [file pcbi.1011753.s002.pdf]

Reviewer 1

In the current manuscript, the authors present a new method to predict the abundance of DNA fragments in asynchronously growing cultures. They use this approach to successfully infer the origins of replication in budding yeast, showing that this method can be used to discover replication origins from sequencing data. The application of their scheme to DNA replication in E. coli shows that it predicts temporal variations in the replication speed with a period that scales with the cell doubling time, suggesting that these variations are related to the cell cycle.

The generic nature of the scheme makes the study very interesting. Moreover, the manuscript is well written and the results are clearly presented. I can therefore recommend publication of this manuscript in PLCB after the authors have satisfactorily addressed the following questions and comments:

We thank the reviewer for her/his appreciation of our work and for recommending publication in PLCB.

For yeast, the authors clearly state that they assume that the firing rate is constant in time. But what do they assume for E. coli? I presume they assume all origins fire once per cell cycle. But do they include stochasticity in the precise moment of firing? And can they use their method to infer (correlated versus uncorrelated) variations in the times at which the respective origins fire? If so, what would their analysis predict for the time between the firing of the first and last origin? Skarstadt et al. have measured this to be on the order of 3 - 4 minutes (Skarstsadt et al., EMBO J., 1986; see also Berger and Ten Wolde, PRXL, 2023). If this inference is possible, I presume the authors could also convolve the variations in the timing with the evolution of the volume with time, to predict variations in the initiation volume. Would that agree with the variance in the initiation mass as measured experimentally (Wallden et al., Cell, 2016; Si et al., Curr. Biol. 2019; Boesen et al. bioRxiv:2022.09.08.507175 (2022)?

We thank the reviewer for this important comment. Our approach provides an ensemble view of DNA replication that is insensitive to the "absolute timing" of the replication program, i.e. the time at which each genome starts being replicated. One way of seeing this is that replication programs that started at different times respect to the cell birth moment, or having different variations around the mean initiation time, would yield, according to our theory, the same DNA abundance distribution, up to a multiplicative constant. This multiplicative constant is hard to determine experimentally, as the normalization of $\mathcal{P}(x)$ depends on experimental details such as the amount of sequenced fragments. On one hand, this is a limitation of our method. On the other hand, this can be seen as an advantage, as our method permits to precisely study the DNA replication program per se, without worrying about its link with the cell cycle.

Thanks to the reviewer's comment, we realized that this point was not sufficiently explained in the manuscript. We therefore added a more thorough discussion of this point at the end of the General Theory section, including the references mentioned by the reviewer.

After an origin has fired, there is typically a refractory time that prevents these origins from rapid refiring. How does that affect the results? Certainly

for E. coli at high growth rates, with cell doubling times of 20 - 30 minutes, the refractory time, of order 10 minutes, is relatively large.

A related question: Which assumptions could be relaxed or introduced, to improve the agreement with experimental data? For example, would imposing a refractory time or stochasticity in the firing moment improve the agreement with experimental data?

We thank the reviewer for these comments. The answers to these points is related with our previous comment. Our theory depends only on the rate of replication initiation in the population, which grows proportionally to the population size. The precise timing between subsequent rounds of replication does not play a role in our population-level framework. We now discuss this point at the end of the General Theory section as well.

Could the authors expand their discussion of the origin of the variations in replication speed. In their previous manuscript (eLife, 2022), they suggested it is related to variations in the mutation rate. Does the current analysis still support that idea? I presume the observation that the period of the speed variations scales with the doubling time, rather than spatial position, provides evidence against this hypothesis? A more detailed discussion would be appreciated.

We thank the reviewer for this comment. Our results suggest that these two phenomena are caused by a temporal, rather than spatial, mechanism. The fact that a temporal mechanism can cause spatial variations may be simply due to the fact that replisome trajectories appear to be nearly deterministic, i.e. their Peclet number is large. In this respect, the result in our manuscript do not provide evidence against a link between variations in speed and mutation rate. We added a few sentences in the Discussion addressing this important point.

Minor Comments

It would help to explain $f(x,\tau)$ in the caption of Fig. 1.

We added a sentence reminding the reader the definition of $f(x,\tau)$.

It would help to explain $\psi(x,\tau)$ in words, the first time it is introduced, i.e. above Eq. (3).

We added a sentence as requested by the reviewer.

Reviewer 2

The authors propose a general theory for extracting information about the DNA replication program from DNA abundance data. The theory is based on a few fundamental assumptions that are valid in most situations, and some of them can potentially be relaxed. The theory begins with a stochastic setting but also generates analytical results for the deterministic limit. One of the advantages of this theory is its applicability to both eukaryotic and prokaryotic cells. It is demonstrated that in eukaryotic cells, the theory can be used to estimate the number and locations of origins, while in prokaryotic

cells, it can be used to calculate variations in the replication speed of the two replisomes. Predictions from the theory align well with the data in both cases. Due to its generality, this theory has the potential to be exploited in future studies on DNA replication in various species.

We thank the reviewer for her/his appreciation of our work and the constructive feedback.

About E. coli. replication, the paper assumed that the replication terminates once the two replisomes meet each other. However, replication termination should be determined by terC where both replisomes stop. How would the result be different if they made this more realistic assumption?

We thank the reviewer for this comment. We have now added a figure to Appendix F that compared our model with simulations that take the tus-ter system into account, and a brief discussion of the results. The simulations show that, for the estimated diffusivities, the effect of the tus-ter system on the abundance curves is negligible. For the effect to become noticeable, the diffusivity has to be increased at least $10\times$ over the estimated value. We mention this additional result in the revised Discussion.

Minor comments

Line 184: what is the indication of a 7 kb binning length scale? Are there references to this length scale?

We thank the reviewer for this comment. The length scale of 7kb is where the correlation between inferred and known origins peak, which provides an estimate of the spatial precision with which origin locations are recovered. We recognize that this was not explained well in the original manuscript; we have now reworded the relevant part of the section *Inferring yeast replication origins from experimental data*, and have added further details on how the correlation is computed in the caption of Fig. 3.

In Eq. (11), the speed modulation function $h(\tau)$ is assumed to be the same for the two replisomes. However, since the sequences that the two replisomes replicate are not symmetric, $h(\tau)$ can be different. In this case, do the two replisomes' speeds strongly correlate with each other? Also, in Conclusions, the authors speculate that the competition between the two replisomes can be a source of speed fluctuation. How would that affect $h(\tau)$ in the model?

We thank the reviewer for this comment. In our previous Elife paper, we assumed the speed fluctuations to be position-dependent. However, the observed fluctuations appear to be remarkably symmetric for the left and right replichores, suggesting that sequence-specific effects do not play an important role. This also suggests a temporal, rather than spatial mechanism underlying the fluctuations. If the mechanism is temporal, as assumed in our manuscript, it is natural to assume that the two replisomes are equally affected. In the revised version, we made this assumption more explicit.

Our hypothesis is that competition between *multiple* replisomes could be the source of speed fluctuations. This hypothesis would predict that the

period of oscillations should be on the order of the cell division time, as origins are fired once per cell cycle. This hypothesis is therefore supported by the data collapse shown in Fig. 5c of the manuscript.

There are similarities between the E. coli. results in this paper and their 2022 Elife paper (https://doi.org/10.7554/eLife.75884), including the model itself, part of the figures, and the main conclusions. It would be worth emphasizing the new information this paper provides.

The bacterial model proposed in this work is different from that in the Elife paper. One main differences is that we postulate a temporal, rather than spatial oscillatory mechanism. A second difference is that, in our manuscript, stochasticity originates from a stalling mechanism, whereas in our previous Elife paper stochasticity was modeled as simple diffusion. A further element of novelty is that we provided an exact solution for this model, which leads to considerably simpler and more robust fits to experimental data.

We do however agree that the main message that we obtained from this model is consistent with that of the Elife paper. This is in a way a good thing, because it shows that the main biological conclusions are robust with respect to the modeling choice. Moreover, we feel that one of the main elements of novelty of our work is to show how this model can be placed in the bigger picture of a general theory of DNA replication, which encompasses bacteria and eukaryotes.

In the revised version, we explain more clearly the elements of novelty of the E.coli results presented here compared to our previous work.

Reviewer 3

This paper presents an original approach to the study of DNA replication, with applications to both E. coli and budding yeast. The focus is on the inference of origin locations as determined from populations of cells that grow asynchronously, using experimental data based on deep sequencing (counting local DNA content). The fact that the data comes from asynchronous cells is significant because such experiments are not only easier to do than ones that attempt to synchronize cell populations, they also avoid various experimental artifacts that arise as a result of direct synchronization. Synchronization can also be done by sorting (FACS and related techniques) but is also only approximate.

The approach taken here generalizes previous work on analysis of DNA replication in bacteria that accounts for the possibility of re-replication and, most impressively, presents a unified framework that is demonstrated to be relevant for both bacterial and eukaryotic replication data. I found Eq. (9) a particularly pretty result. And the derivation of Eq. (6) is much more straightforward than the one previously given in Ref. [22].

Although I have a number of main and minor comments, I am overall very impressed by this paper and think it makes a significant contribution. Many of the comments are either to clarify what has been done or to press a bit harder for what might be easy generalizations.

We thank the reviewer for her/his appreciation of our work.

The title is a bit off, in that 1/3 of the text concerns eukaryotic replication and indeed, the authors claim that the theory they develop "equally applies to bacteria and eukaryotes" (L49). A title that included both cases would more accurately reflect the contents of the paper. I think this is important because this paper is perhaps unique in advancing a description of DNA replication kinetics that is demonstrated to be relevant for both prokaryotic and eukaryotic organisms.

We thank the reviewer for this comment. We appreciate the suggestion, but after giving the matter some thought we prefer to keep the title as it is. This might be a matter of taste, but our feeling is that a relatively short and simple title better reflects the generality of our approach. Moreover, the term "microbial" does in principle include unicellular eukaryotes, and the fact that we study both bacteria and eukaryotes is made explicit in the abstract.

Please comment more about the way stalling is modeled. I get that Eq. 11 is a convenient way for having a process with stalls, but it gives a temporal stochastic mechanism whereas previous discussion of stalls tends to view it as location specific. See, for example, the discussion and related references in Ref. [22]. Does this difference matter? Also, please elaborate on the properties of the stalls. What is the distribution of stall durations? Using the Péclet number to assess the significance of stalls seems plausible, but can one justify it more precisely? Finally, does this discussion have anything to do with the $v = v(x)$ *or* $v(t)$ *discussion in the paper / SI?*

We thank the reviewer for this comment. The stalling discussed in Ref. [22] is in the context of Bacillus subtilis, where replication stalls at a well-defined point. Such deterministic, well-defined points of replication stalls do not seem to be present in our experimental data for *E. coli*, nor in those of Ref. [22]. Our model captures a fundamentally different kind of stalling, being stochastic and location-independent. We clarified this conceptual difference in the revised manuscript. We agree with the reviewer that it would be interesting to study the statistics of stall duration and compare it with experimental data. We mention such perspective in the revised Discussion, mentioning a study that performed a similar analysis for an RNA polymerase.

How sensitive is the model and analysis to the assumption of perfect asynchrony? How could one assess that asynchrony experimentally? The authors refer to this issue briefly but somewhat opaquely in Appendix A, after (A2). I didn't really follow their comments there. For example, can one relate the growth function to a distribution of cell-cycle "phases"? And, again, can one determine from the data the form of that function?

We thank the reviewer for this comment. In the case of non-perfect asynchrony, one expects to observe an exponential growth modulated by oscillations. The formalism of Appendix A permits to deal with these cases, but, in practice, experimentally quantifying these oscillations can be daunting. In any case, we have shown in our previous work that, for common experimental protocols, the effect of non-perfect asynchrony can be safely neglected. In the revised manuscript, we expanded Appendix A to address all of these points.

Minor Comments

Author summary: infer the origins of replication in budding yeast [there are more than one]

We thank the reviewer for pointing out this. We changed the text accordingly.

Line 13: "some origin sites may not be activated at all" makes it sound like this is a rare occurrence. But in budding yeast, more than half of the origins are not activated in a given cell cycle.

We thank the reviewer for pointing out this. We reworded the sentence.

Lines 90–95: the definition of mathcalP(x) is not as clear as it might be. In particular, what do the authors mean by "randomly chosen genome"? As usually defined, genome includes all locations x, so the sentence makes it sound like mathcalP(x) = 1, by definition. Since that isn't what is meant, the sentences should be written more carefully, and perhaps have a sketch to illustrate.

We thank the reviewer for the comment. We have addressed this by emphasizing that "genomes" include incomplete genomes that are undergoing synthesis.

Line 122: can not → cannot

We made the suggested change.

Line 179: The authors mention that they infer 234 origins. This would be a good place to mention that K=354 were found in the original study and to briefly discuss. (The number is buried in the caption to Fig. 3, but it seems natural to discuss here.)

We thank the reviewer and agree with her/his suggestion; the text now states the number of known origins as well.

Fig. 3 and surrounding discussion: Please give more details on the correlation measurement (define the quantity presented). Also, please be more precise in what was done for the shifted and shuffled surrogate data sets.

We thank the reviewer for making us aware that our description lacked details. We have expanded the figure legend to explain the shifting and shuffling in more detail, and the method by which we compute correlations.

For the Langevin equations (11), please discuss more why it is appropriate to include the modulation in both the velocity and the noise strength.

We thank the reviewer for this comment. We made this assumption mostly because of mathematical convenience, as it considerably simplifies solving the model. We note that the impact of this assumption is minimal in practive, as the diffusion constant D is very small and the function $h(\tau)$ is characterized by small variations around $h(\tau) = 1$. In the revised version,

we clarified that this assumption is mostly motivated by mathematical convenience.

Line 232: smoothen → smooth
Fig. 5 caption: Bacterial model fitted → Bacterial model fit

We made the suggested changes.

Line 296: In discussing the generalization to variable initiation rates, in addition to Ref. [10], it would be good to refer to A. Goldar et al, "Universal Temporal Profiles of Replication Origin Activation in Eukaryotes", PLoS ONE 4, e5899 (2009).

We thank the reviewer for suggesting this reference. We added it to the bibliography.

Lines 311–312: "fork firing". Do the authors mean origin firing? (In the usual language, the origins fire, creating two forks that then move or "progress".)

We made the suggested change.
